# Repositories for academic products/outputs: Latin American and Chilean visions

**DOI:** 10.12688/f1000research.19976.1

**Published:** 2019-08-28

**Authors:** Leandro Torres, Ricardo Hartley

**Affiliations:** 1 Instituto de Investigación e Innovación en Salud, Universidad Central de Chile, Lord Cochrane 417, Santiago, 8330507, Chile; 2 Instituto de Ciencias Naturales, Universidad de las Américas, Av. Manuel Montt 948, Providencia, Santiago, Chile

**Keywords:** Repositories, Chile, Latin America, Scientific Data, Open Access

## Abstract

Open access policies have been progressing since the beginning of this century. Important global initiatives, both public and private, have set the tone for what we understand by open access. The emergence of tools and web platforms for open access (both legal and illegal) have placed the focus of the discussion on open access to knowledge, both for academics and for the general public, who finance such research through their taxes, particularly in Latin America. This historically unnoticed discussion must, we believe, be discussed publicly, given the characteristics of the Latin American scientific community, as well as its funding sources. This article includes an overview of what is meant by open access and describes the origins of the term, both in its philosophical sense and in its practical sense, expressed in the global declarations of Berlin and Bethesda. It also includes the notion of open access managed (or not) by some reputable institutions in Chile, such as CONICYT (National Commission for Scientific and Technological Research) and higher education institutions reputed nationally, such as the Universdad de Chile and Pontificia Universidad Católica de Chile. Various Latin American initiatives related to open access (Scielo, Redalyc, among others) are described, as well as the presence of Chilean documents in those platforms. The national institutional repositories are listed, as well as their current status and a discussion about what open access has implied in Latin America and its importance for the replicability of the investigations carried out locally. Finally, we describe some governmental initiatives (mainly legislative) at the Latin American level and propose some recommendations regarding the promotion and implementation of repositories for the access to scientific data (for access and replication purposes) of the national research.

## Introduction

Internationally, open access (OA) policies have gradually advanced in academia and beyond. Well known are national level OA policies and regulations from Peru ^1^, Argentina ^2^ and Mexico: Decreto Ley ^3^, as well as global initiatives to remove obstacles for the access, distribution and re-use of academic research outputs in institutions that fund research, such as the National Institute of Health (NIH) ^4^ and the National Science Foundation (NSF) ^5^ in the United States, philanthropic organizations like the Bill and Melinda Foundation ^6^, the European Commission ^7^ and the Wellcome Trust in the UK ^8^, among others (further information about OA at global level can be found in 9).

Another important element in the promotion of OA is the development of tools that can accelerate this process, that can direct to publications stored in OA repositories, such as Science Open, 1Science and web browser extensions such as Canary Haz ^10^ and Unpaywall ^11^, and the controversial academic social networks ^12^ that allow self-archiving and distribution of publications such as academia.edu ^13^ and researchgate ^14^. This latter is currently under legal scrutiny for distributing academic publications ^15^.

In the case of Latin America, while there has been important work in pushing some initiatives, some of which have shown progress, results do not seem very encouraging ^16, 17^. According to recent publication statistics, there is a linear increase in publications in OA journals; this is, however, rarely contrasted with the increase in the number of publications in non-OA journals in the region during 2013–2017, a trend that appears to be growing, at least according to indexes such as Web of Science. Therefore, the growth of OA has not had an effect on the number of publications in paywalled journals; instead, we see an increase in both and especially the latter.

In this analysis, it is necessary to take into account that OA refers only to documents that have been made available through ’Gold’ and ’Green’ OA modalities ^18^. The former refers (although not in all cases) to OA articles for which publication is associated with article processing costs (APCs), while the latter makes reference to self-archiving, in which case a version of the peer-reviewed article is made available through an online repository or website, and archiving will depend on the policies that each journal imposes ^19^.

OA has been characterized by various authors, who have described its different varieties, among which we can identify: Libre OA ^20^, Gratis OA ^20^, Gold OA ^21, 22^, Green OA ^23^, Hybrid OA ^24^, Delayed OA: ^25^ and Bronze OA ^26^.

In addition, we should consider the following two non-traditional alternatives: Academic Social Networks (ASN), for-profit social networks that allow academics to share their publications, with more than half of their content being shared illegally; and Black OA, articles shared on illegal pirate sites such as SciHub. The data that Sci-hub has provided with respect to downloads from Chile (February 2016) show that these download concentrate mainly in the capital Santiago, where 273,834 articles were downloaded, followed by Concepción (31,985), Valdivia (22,069), Valparaíso (16,075) and Viña del Mar (17,024) ^27^.

**Academic Social Networks (ASN):** corresponds to for-profit social networks that allow academics to share their publications. Although some include OA definitions ^12^, others consider that the content shared through this platforms is not OA since, in contrast to Green OA repositories, these do not check copyrights and therefore, more than half of the its content is stored and shared illegally ^28^, causing controversy ^29^.

**Black OA:** corresponds to articles shared on illegal pirate sites, mainly Sci-Hub and LibGen ^29^.

Related to Black OA, the data that Sci-hub has provided with respect to downloads from Chile (February 2016) show that these download concentrate mainly in the capital Santiago, where 273,834 articles were downloaded, followed by Concepción (31,985), Valdivia (22,069), Valparaíso (16,075) and Viña del Mar (17,024) ^27^. Access to content through this kind of OA is common, as if all academic publications worldwide are considered, only 25% are disseminated through any existing form of OA, not including ASNs and black OA ^30^.

In Latin America, historical OA movements such as SciELO, CLACSO, Redalyc and, more recently, LA Referencia, form the basis of what is understood by OA in the region. It has been argued that what “characterizes the Latin American flavour of OA” is that journals that constitute it are supported by universities, research institutes and other academic organizations without APCs ^31^. However, obstacles in the interoperability between different search engines such as SciELO, Redalyc and LA Referencia ^31^ hinder analyses of the necessary metrics that would allow a clear perspective on the advancement of the OA movement in the region. We must consider that, at least in Chile, the cost of collection subscriptions to publishers such as Elsevier, Springer-Nature, Wiley, American Chemical Society, Annual Reviews, Oxford University Press and AAAS from 2008 to 2017 was $95,754,011 USD for universities, government and other educational institutions, according to Consorcio para el Acceso a la Información Científica Electrónica, ^32^, making it possible to reduce the cost of access to each article from $20 USD to $3.

Despite these initiatives, out of the 20 countries considered part of Latin America, only Argentina, Perú and México have national laws that promote OA, especially through the development of institutional repositories (Perú ^1^), Argentina ^2^, México: Decreto Ley ^3^), while others such as Colombia, Brazil and Chile have focused mainly on the management of national systems of digital repositories, despite the absence of mandatory policies for OA in national research.

## OA, a general vision

The philosophical roots of the information policies expressed as OA have their origins in the philosophy known as ’open society’, proposed by Henri Bergson (1859–1941), Karl Popper (1902–1994) and George Soros (1930), that propose fostering values such as freedom, progress, equality, fraternity, tolerance, rejection of tyranny, censorship and the exercise of power as a form of control.

The creation of institutions such as the Open Society Institute by George Soros (the name of which is inspired by the book ’Open Society and Its Enemies’ by Karl Popper, published in 1945), provided the context in which initiatives such as the Budapest Open Access Initiative ^33, 34^, the Berlin Declaration on Open Access to Knowledge in the Sciences and Humanities (2003) ^35^ and Bethesda Statement on Open Access Publishing (also from 2003 and more oriented to research in the natural sciences) ^36^ emerged.

In general, OA terms and (maybe even more importantly) access to research outputs, generally in the form of scientific publications, mean that social agents besides researchers are able to interact with such outputs, both in sciences and humanities. This democratization of knowledge creates a problem of access: it is not enough to make just the content available, it should also be interpretable by any of those who see it. It is maybe at this point that knowledge moves from being ’visible’ to being ’accessible’.

Although this might seem obvious, it is of particular relevance if we consider that many of our individual decisions, as well as our collective political decisions, are many times based (or at least, should be based) on the knowledge that we have about certain phenomena. This point will be discussed later, when we reflect about what is understood by ’public good’, a fundamental element that often justifies the adoption of OA policies.

## The vision of OA in Chile

In order to have a clear perspective of what OA entails at national level, we will take as reference the definitions of OA used by institutions associated with research in sciences and the humanities, taking as an example CONICYT (the largest funding body of science in Chile), Universidad de Chile and Pontificia Universidad Católica de Chile (both traditional universities and with high publication rates).

The first of these organizations is the main agency that funds and regulates academic research in Chile, and the other two are the most prestigious universities in terms of academic and research performance in the country, (highest QS ranked Chilean Universities in 2019).

To our knowledge, neither Conicyt nor Pontificia Universidad Católica de Chile have a clear definition of what is considered as OA; however, Universidad de Chile states on its website ^37^:

- “The Open Access Movement is an initiative that promotes free access to digital materials derived from scientific or academic production, beyond copyrights, that these materials may hold.”
- “The idea is to enable people to read, download, copy, distribute, search or link these resources without needing to register or pay. This access is often performed through the internet and for this reason, the authors of these materials will receive a higher dissemination of their work. These digital contents can include articles published in online journals, images, data, audiovisual material, and any other digital content whose author wishes to give free access”.

To comply with these objectives, they propose options to publish in OA journals, among which they include the 5000 magazines present in the Directory of Open Access Journals (DOAJ), and institutional repositories such as Universidad de Chile’s repository CAPTURA, CONICYT’s repository and SHERPA/RoMEo ^38^.

Willisky ^39^ and Fischman ^40^ propose 10 different ’flavours’ of OA that correspond to different strategies oriented towards the diffusion and promotion of academic knowledge. Those correspond to 10 different “flavours” of Open Access are: Home page (websites in which a profile of the academics and their publications are made available), E-print (academic repository that allows self archiving published and unpublished material), Author fee (fees support to immediate and complete access to journals or individual paid articles), Subsidized (different institutions enable complete access to open access journals), dual mode (there is a subscription to the print edition that is used to support the digital and printed formats), Delayed (subscription fees are collected for immediate access and to sustain a printed edition, the content is fully available after a period of time), Partial (a part of the content is open access whereas the rest require a subscription). These flavours are specially used by publishers from developed countries to promote OA. In contrast, developing countries as Chile usually adopt Per capita subscriptions (expense limited to registering institutions in an access management system, such as Cincel and others) and Indexing (access to abstract and bibliographic information provided as a government service, with access to whole articles obtained by paying per view). Finally, a certain amount of the academic production in Chile is supported by academic institutions affiliated to SciELO Chile. This last modality is known as ’Cooperative’.

## Latin American OA initiatives and making Chilean research visible

**Latindex (Latin American Index of Serial Scientific Publications):** a system for online dissemination that gathers information about scientific research journals, professional magazines and magazines dedicated to scientific and cultural outreach, from countries in Latin America, the Caribbean, Spain and Portugal. It emerged as an initiative from the National Autonomous University of Mexico (UNAM) in 1995, becoming a network for regional cooperation since 1997. It offers data related to print or online journals that comply with academic criteria (in a broader sense) of quality that Latindex has established, as well as access to full academic journals and articles in the different languages used in Latin America. It is the most inclusive source ^41^, since from its origins it used files present in the platforms CLASE, PERIODICA and LILACS, although currently it has a broader coverage. For Latindex, the identification, registry and update of its entries has been difficult due to the different criteria and standards for publication. However, it has been able to gather fundamental data about publications in the affiliated regions. In 2018, Latindex registered 26,010 journals, of which 15,948 are Latin American (South America, Central America and the Caribbean). Of those, 2,147 are Chilean, which represents 8,251% of the total; among these, 2,038 are still being issued, 105 are not being published anymore and four have an unknown status ^41^.

Latindex provides users access to a directory that takes into account traditional journals with an international distribution as well as newer journals with limited distribution. The criteria to be included in the directory are the following ( https://www.latindex.org/latindex/regRev): The journal must be at least two years old, item The official or institutional website of the journal will be assessed and must have free access to all its contents, non one pdf journal, among others.

**SciELO (Scientific Electronic Library Online):** a scientific library with a digital database of academic articles belonging to different disciplines, freely available in full text, which allows for preparation, outreach, storage and evaluation of academic literature in electronic format. It works under a set of common criteria of publication and software, and its operation is based on national collections of academic journals with editorial committees and is peer reviewed. One of its main purposes is to make visible academic articles in local languages (Meneghini 2006). By 2018, it indexed 1,285 journals, 145,182 academic articles and 16,943,454 citations (confirma SciELO en números). This initiative was developed by São Paulo Research Foundation (Fundação de Amparo à Pesquisa do Estado de São Paulo FAFESP) and the Latin American and Caribbean Center on Health Sciences Information (BIREME). SciELO Chile indexes 121 journals, of which 107 are still being issued and 14 are no longer published. Each SciELO website is responsible for the costs and responsibilities of obtaining citation data of the indexed articles. In some cases, this is performed by the academic journals and in others, the organization in charge of SciELO ^42^.

**Redalyc:** a more recently created indexing and publication platform. This network publishes more than 1,278 academic journals from Ibero America and the Caribbean. It stores about 47,056 individual issues and 609,283 full text articles and documents of diverse nature, being the database that provides the largest amount of metadata associated to authorship and co-authorship in Latin America. With respect to Chile, RedALyC indexes 93 journals, 37,921 full articles and 110 instalments. The catalogue of RedALyC selects journals in the social sciences and humanities based on a set of criteria (for details see 43). Despite its high level of access to academic documentation, RedALyC still lacks a citation processing system and impact indications in the region. Its broad access to journals in the region has allowed it to harvest a large amount of metadata associated with publications, which represents an important potential source for analysis in issues related to academic collaboration both inside and outside the region, using what they refer as “production profiles” ^44^.

**CLACSO (Latin American Council of Social Sciences):** a non-governmental international organization created in 1967 in which 47 countries participate (including United States, Canada, Germany, France, Portugal and Latin American countries) with a total of 616 research centres in the social sciences and the humanities. It has generated initiatives that promote the development of OA in different regions through the publication of books, journals and other formats through its virtual library ^45^, the Latin American and the Caribbean Library of Social Sciences and CLACSO TV ^46^.

**LA Referencia:** The Federated Network of Institutional Repositories of Scientific Publications, LA Referencia, was created on the 29 of November, 2012, after the signature of a cooperation agreement in Buenos Aires ^47^, with the purpose of providing OA to academic research financed through public funds in the region. It is a network of OA repositories present in nine countries (Argentina, Brazil, Chile, Colombia, Ecuador, El Salvador, Mexico, Peru and Costa Rica). This platform aims to generate an interoperable system, in which it would be possible to share and make visible academic outputs generated in higher education institutions and scientific research organizations. It is operated by national nodes, storing and hosting academic articles and post-graduate theses. It is based on agreements between public agencies of science and technologies (ministries and national agencies of science and technology) of the member countries, together with red-CLARA (Latin American Cooperation of Advanced Networks), whose objective is to connect academic computational networks in Latin America ^48, 49^. Its purposes include (1) to realize regional agreements to define common standards and policies, (2) to ’harvest’ the registries of metadata obtained from each national node, (3) to validate the quality of the data obtained, (4) to generate a unified search service (federate) and (5) to facilitate access to full text documents and bibliometric data ^50^. The national node corresponding to Chile has a council of directors belonging to the National Commission of Scientific and Technological Research (CONICYT), and its function is “to strengthen and ensure the access to national and international scientific information with the purpose of research, education and innovation”, by managing the national infrastructure of access to STI information. The policy and regulation that supports its actions is the ’proposal for open data’ generated by the government of Chile in 2014, which introduces seven relevant aspects that recipients of CONICYT grants should consider, such as: publish data (non mentioning raw data) and other products in institutional repositories, provide the location to CONICYT, publish before one year from final report, CONICYT “should” publish final report not longer than 3 months after the approval.

In this respect, CONICYT provides recommendations for OA and preserving scientific information and data in the ’Manual for open data’ ^51^. As a platform, it is managed by Web SIC (Scientific Information System) that hosts CONICYT’s Digital Repository, in which it is possible to store and access the results of research, productivity and instruments financed by this agency. The Chilean institutions that participate in LA Referencia can be found on SciELO-Chile and their Institutional Repository.

Standardization, regional and international interoperability, regional collection of data and training are some of the main objectives of this initiative, that work together with The Confederation of Open Access Repositories (COAR) ^52^.

## Chilean initiatives: OA repositories and journals

The Chilean academic heritage is published and preserved through national efforts of different kinds of institutions, such as universities and government organizations. The efforts have been directed towards the construction of institutional repositories (IRs) and journals that feature OA licenses (OAJ). These store, share and provide access to academic products from institutions and their researchers. This research can be made available in various formats, from theses, monographs and preprints to published documents (in OA journals or with distribution licenses).

Three important initiatives have emerged as a way of organizing and making visible global OA content: Directory of Open Access Repositories (OpenDOAR, lists OA repositories), Directory of Open Access Journals (DOAJ, where OA journals are indexed) and Registry of Open Access Repositories (ROAR, indexes the growth of institutional OA repositories and OA policies).

In ROAR ^9^, Chile registers 22 entries, of which 16 belong to higher education institutions, one is SciELO Chile, one the Digital Library of the Museum of Memory and Human Rights and one the digital repository of CONICYT. The rest of the repositories correspond to duplicated or inactive entries.

Of the 22 repositories, 13 correspond to institutional repositories of Chilean universities, four correspond to institutional repositories of government bodies and 13 operate using the DSpace software for the creation and management of digital repositories.

In DOAJ, Chile registers 113 OA journals, most of them corresponding to publications related to the social sciences. Among these, 109 do not charge APCs. With respect to licenses, 39 use the licence CC-BY, 26 the licence CC BY-NC-ND, 17 use the license CC BY-NC-SA, 15 the licence CC BY-NC, 12 the licence CC BY-SA (12), and only three use specific editorial licenses (further details about the editorial licenses can be found in 54).

Despite the high number of institutional repositories, it seems as if there is no explicit recognition from the authorities and national organizations regarding academic research in relation to this matter, since there are no mandated policies with respect to the need, construction and implementation of this type of repository.

**Table 1.** Chilean institutional repositories (Source: 53).

N°	Name of the RI	Type of RI	Software that supports it
1	Digital Library of the Museum of Memory and Human Rights	Disciplinary	Greenstone
2	Digital Library of the Centre of Information on Natural Resources (CIREN)	Institutional	DSpace
3	Cybertesis Pontificia Universidad Católica de Valparaíso	Institutional	Cybertesis
4	DSpace Universidad de Talca	Institutional	DSpace
5	Difunde	Institutional	DSpace
6	ECLAC Digital Repository	Disciplinary	DSpace
7	Digital Books Website - Universidad de Chile	Institutional	Other (Open Monograph Press)
8	Website for Chilean Electronic Thesis	Institutional	Other (Open Harvester System)
9	Website for Latin American Thesis	Institutional	Other (Open Harvester System)
10	Academic Repository of Universidad de Chile	Institutional	DSpace
11	Digital Academic Repository of UC Temuco	Institutional	DSpace
12	Digital Repository Conicyt Rl 2.0	Institutional	DSpace
13	Institutional Academic Repository Universidad Andrés Bello	Institutional	DSpace
14	Institutional Repository Universidad del Desarrollo	Institutional	DSpace
15	Repository UC (Pontificia Universidad Católica de Chile)	Disciplinary	Greenstone
16	Repository Universidad de Concepción	Institutional	DSpace
17	Academic Repository UTEM	Institutional	Cybertesis
18	Scientific Electronic Library Online – Chile	Aggregating	SciELO
19	Electronic Thesis UACh	Institutional	Cybertesis
20	Electronic Thesis from Universidad de Chile	Institutional	DSpace
21	Universidad del Bío-Bío Cybertesis Library Network	Institutional	Cybertesis
22	Virtual Library of Cieplan	Disciplinary	Software Other (HTML)

## The implications of worldwide OA and how has it been understood in Latin America: the importance of replicability and reproducibility of science

As mentioned above, and according to the Budapest Declaration, OA is defined as an editorial model where access and use of scientific literature is free ^55, 56^). In Latin America, there are various interpretations of the meaning of OA and the consequences of accepting some of these meanings, due to different motivations for participation in this movement ^57, 58^. Whatever its meaning for the organizations in charge of this initiative (national science and technology agencies, universities, editorials, etc.) in Latin America, it is clear that currently, two ways of OA are recognized: ’Green OA’, in which the papers that have been published under a traditional model or that are in the process of being published are made available in repositories; and ’Gold OA’, in which articles are published in journals that have ’opening’ mechanisms in their publications, generally with publications costs for the researcher or its founders. In either case, the method of access is through the internet.

The impact of both methods has been recently tested and it was been concluded that only 13% of the articles published between 2008 and 2015 are available through Green OA, while 9% were available through Gold OA, showing that most of what is being published is being hidden under a ’paywall’ ^59^, making this knowledge practically inaccessible to researchers and institutions that do not have large funding sources and even more difficult in the case of access for educational purposes and the general public.

While so far we have stressed the importance of OA for citizens, an important aspect, and probably essential for science itself (and the humanities) ^60– 64^ is the possibility to critically analyze the corrections of scientific declarations and the conclusions that other scientists have reached and their data analysis, both at systematic and statistical levels ^65^). To fulfill this task, it is necessary that other researchers can reuse, replicate and reproduce the findings reported in scientific publications. This terminology has been a source of confusion (to learn more about this confusion, see 66). Goodman ^67^) proposes the following definitions of reproducibility:

**Methods reproducibility:** provide sufficient detail about procedures and data so that the same procedures could be exactly repeated.

**Results reproducibility:** obtain the same results from an independent study with procedures as closely matched to the original study as possible.

**Inferential reproducibility:** draw the same conclusions from either an independent replication of a study or a reanalysis of the original study.

To achieve reproducibility, it is necessary that researchers share materials, protocols and data that sustain findings, with the purpose of confirming previous studies or becoming the basis for new studies. For example, Nature Cell Biology ^68^ proposes that the minimum combination of data would include not only the data presented in the publication itself, but also the unprocessed numerical data that underlie the graphs and quantitative evaluations, the independent repetitions of representative experiments that provide the support for reproducibility in the results and sets of large scale data generated by the study ^69^.

These data should be available with the published document (including the original data without processing and independent repetitions). However, larger datasets could be stored in public databases and their location specified in the corresponding document. In the absence of specific OA databases (for example, for new techniques or for which there is still no public database), Nature recommends to store data in general data repositories, such as Figshare ^70^ or Dryad ^71^. This databases can also be complemented, depending on its appropriateness, with tools like Zenodo ^72^, Github ^73^, Gitlab ^74^ or OpenScience Framework ^75^, among others, as platforms for publication, as well as for preprints, data and supplementary code resulting from a research project.

Despite the relevance of OA databases, a study performed in 318 biomedical research ^76^ journals found that, as reflected in the instructions for authors and their editorial policies, only a small percentage (11.9%) declare explicitly that sharing data is a necessary condition for publication, 9.1% requested shared data (without this being an explicit condition for publication), 23.3% of journals promote sharing data but not as mandatory, 9.1% indirectly mention the relevance of sharing data and 14.8% propose to share data of proteins, proteomics and genomics. The 31% left does not mention data sharing practices. Although, as the authors mention, 65,7% of the journals require data sharing as a criteria for replicability, they do not provide specific policies or guidelines about these practices that could ensure that the data is available and reusable.

In Latin America, and in particular in Chile, little emphasis has been given to the importance of access to data or the development and promotion of platforms to store this type of research output with the purpose of promoting reproducibility. A Latin American study analyzed whether there are differences in the beliefs that regulate research between natural and social scientists ^77^. The article examines the social agreements that regulate the behaviour of academic researchers through an opinion survey in which 185 active researchers participated (of which, 96% declared to have completed postgraduate studies).

This study proposes four fundamental (intuitive and informal) agreements that constitute scientific work, including:

- The world has laws or regularities that are understood through observation
- You must have the ability to analyze in an objective, impartial, verifiable and systematic way the information provided by reality ("having a critical attitude").
- Have mastery of technical aspects of the work, the correct use of the recording devices, the baseline calibration for each experiment, the design of appropriate controls ("have methodological aptitude"), among others.
- The results should be communicated in an open way, that is, verifiable or replicable.

The most remarkable element of this work in terms of OA (as a means of communication of science) is precisely that expressed in the last point, since the interviewees (96% natural scientists 91 social scientists) mention that the importance of ensuring the veracity and verifiability of the data reported by an investigation are necessary for the advancement of knowledge.

Although this research does not discuss how to achieve reproducibility through the use of OA databases, it is one of the few precedents in this topic in Latin America. Additionally, in some blogs on the SciELO website, the problem of replicability has been superficially discussed. In these blog entries, Lilian Nassi-Caló ^78, 79^ discusses what has been going on with the so-called ’reproducibility crisis’ of certain scientific disciplines, but makes no mention to what this implies for the future of Latin American science. The only claim that CONICYT has made on this topic was to define what is understood as scientific knowledge, including in this definition the idea of reproducibility (without a deeper discussion of the concept):

“Scientific knowledge, generated in this way, has the qualities of reproducibility and objectivity. Precisely herein resides an essential part of its enormous utility, since its predictive value applies to all situations in which the established conditions are reproduced, despite the subjectivity of the observer” ^80^.

As mentioned, Chile does not have clear perspectives or statements about public databases and their relevance, not only for OA itself, but also for reproducibility of Chilean and international science, since it is possible that in the near future, what we today call impact factor will be highly conditioned by the reproducibility of the results of research.

In this light, it seems evident that the exchange of data and its accessibility is fundamental to the advancement of knowledge in the natural sciences, social sciences and humanities, since it allows validation and to contribute with new knowledge about information already published. This supports the development of research and innovation at national and international levels, together with democratization of access to such data or registries in many cases. All this will happen only if this information is available, in an organized and coherent way, according to the declarations of data availability. This can be observed both in prescriptions of governmental mandates, as well as editorials in charge of access and dissemination of the outputs of research. To learn more about this, please see 68 and 81– 83.

## Reflections about the current state of OA in Chile

As mentioned above, OA and the availability of resources are important, not only for the development of new knowledge, but also for social, economic and political advancement in a democratic society. If we only analyze South American countries, according to ROARMAP, only 49 policies have been adopted in this region, of which seven correspond to Argentina, one to Bolivia, six to Colombia, eight to Perú and four to Venezuela. Brazil leads the list with 23 policies ^84^. Most of these policies have been developed by research organizations. Despite Latin American efforts, to date (March 2019), Chile is not present in this database.

As we discussed in this paper, although there are some Latin American initiatives that support OA, mainly in the use of licenses and the development of international databases (Scielo, Redalyc, CLACSO library, LA Referencia), it is important to note that there are still important challenges for interoperability and access to documents (access in terms of a platform or a system enabling the quick and precise location of a document, and interoperability in terms of a systematization of metadata from these different initiatives).

It is important to highlight that university repositories are an essential element in the diffusion and communication of knowledge. The number of digital documents that universities systematically produce, considering academics, undergraduate and postgraduate students, are elements that can help us define the profile of the university, regional, national and international research, and its opportunities for collaboration. This goes far beyond simple university outreach and public engagement activities, and should be treated as a research process in itself. This is why it is an essential element of the academic cycle, implying a responsibility for the administration in the development of technological capacities, as well as human resources within the institution (here, we call it human resources since the type of collaboration required for this purpose is interdisciplinary and does not limit itself to academic titles or degrees, an aspect poorly developed in Chile). This is not a new revelation; in fact, it has been mentioned by the Association of Research Libraries ^85^, among others.

A fundamental role of universities is the dissemination of knowledge and the recognition of intellectual capital and knowledge produced in them. For this, they must develop strategies that guarantee the distribution of the generated content, which implies that they must recover the ability to manage their intellectual capital in order to promote the resolution of local (or global) problems. Therefore, it is important that local governments promote (and finance) these types of initiatives.

The latter is of particular relevance considering the current state of OA worldwide and the recent emergence of Plan S, an initiative developed by cOAlition in 2018 and revised in 2019 and that proposes 10 principles so that in 2021, publicly funded research is published exclusively in OA journals. Several of the principles are ambiguous and inaccurate and some controversial, such as the impediment of researchers to publish in hybrid journals, preventing researchers from publishing in about 80% of academic journals, including Nature, Science, The Lancet, etc. In response to this, more than 1500 researchers, including two Nobel Prize winners, signed a letter arguing against the plan. Agostini and Berk ^85^ point out various points of relevance when considering the current status of Plan S, important elements to be considered by Latin American countries for approval by researchers in Latin American financing agencies, including: (1) quality versus number of publications (the OA business model prioritizes quantity over quality, favoring the appearance of predatory journals, which generates waves of misinformation); (2) increases in the costs associated with publication that could only be financed by governments and richer institutions, generating a gap of inequality; (3) segregation of research quality, diversity and isolationism (countries that generate the highest amount of high quality publications will continue to publish in high-impact journals while researchers from emerging countries will not); (4) would compromise the peer review process (high publication costs would reduce the number of researchers willing to review for “inequality aversion” free of charge, but if the reviewers are paid it would increase the cost for researchers who publish in open journals); and (5) a reproducibility crisis (total open access will facilitate publication with lower quality thresholds, the public would be exposed to a world of information that is more accessible but less accurate and easier to manipulate). In particular for this last point, we point out that the use of models and knowledge management platforms that combine being reader-friendly with a depth of detail that allows critical reading by specialists and non-specialists is required. It is evident that the countries of Latin America will be at a disadvantage in the face of these type of measures, considering that most of the research funding is through public funds. This is particularly important if we think in the little funding (or lack in some cases) to promote open access in South America and the redundancy of initiatives trying to ’harvest’ publications and their metadata and trying to make different platforms ’interoperable’. We propose instead that the development and maintenance of institutional repositories and the use of already existing platforms should be prioritized.

Our proposal aims to promote and strengthen the use of institutional repositories as disciplinary (national and/or global), in parallel to the efforts made to increase the quality of research in the region mainly in strengthening the reproducibility and/or replicability of the investigations developed.

We should also consider what is indicated in the document OpenUP – 7107220 Deliverable D4.3 Good practices and lessons learned. Briefly, research dissemination, as defined by Wilson [ **?** ], facilitates research uptake and understanding. Furthermore, when implementing an institutional repository in Latin America and, in particular, in the Chilean context, it is necessary to explore and focus efforts on the prescriptions provided by institutions with a well-defined trajectory, such as The Repositories Support Project (RSP), an initiative created to “contribute to the creation of capacities, knowledge and abilities in higher education institutions of the United Kingdom” ^84^. RSP has developed a repertoire of considerations to select the best platform to be implemented, taking into account technical aspects ^86^, metadata, repository management, outreach and user engagement and a checklist ^87^.

Also important, on a prescriptional basis, is the use of ’quality seals’ with respect to repositories, such as CoreTrustSeal ^88^, based on DSA-WDS Core Trustworthy Data Repositories Requirements ^89^. These initiatives seek to generate an adequate provision of (1) services for authors and editors (2) deposit, treatment and long term storage of documents and metadata of the objects stored (3) public availability of the objects, which guarantees the possibility of access for humans and machines (necessary for complementary and integral services) and (4) the transference of metadata.

Lastly, we propose a ’Guide for the evaluation of institutional research repositories’ that, together with the directives of OpenAIRE ^90^, considers the following aspects:

- Visibility
- Policies
- Legal aspects
- Descriptive metadata for publication (OAI-DC)
- Logs and statistics
- Security, authenticity and integrity of data
- Services and added value functionality

We should also take into account aspects associated with national and institutional intellectual property policies. Usually, with respect to scientific articles, most scientific journals request the transfer of copyrights to the journal, whose consequences includes the loss of rights to publish of one owns work on a personal website without permission of the editor and the inability to provide copies of one’s own work for distribution and utilization as an educational tool or in the development of academic curricula ^91^. To remedy these problems, an important measure is to modify the agreements provided by journal editors. For this reason, SPARC (Scholarly Publishing and Academic Resources Coalition) has created an Appendix for authors that can be attached to the publication agreement of journals ^92^.

We believe that any institutional repository should allow access, not only to the document in a legible format such as a PDF of the print version, but also should allow access for technologies such as data mining and other free services, which would promote reuse and add value to research already conducted. This could be achieved through the use of repositories that fulfill the minimum requirements to be incorporated in services such as CORE ^93^, which collect all OA content from different sources for its analysis. In this sense, and as a way to generate added value and true access for all citizens that finance, through public funds, these initiatives ^49^, we propose to conduct an exhaustive bibliometric analysis that will allow researchers to establish narratives of conservation, territorial and urban planning, and more. This is not new, but is one of the outreach strategies proposed by Rogers (1962). According to Wilson ^94^, diffusion of innovation “offers a theory of how, why, and at what rate practices or innovations spread through defined populations and social systems". Also OpenUP – 7107220 Deliverable D4.3 Good practices and lessons learned ^95^ should be considered a central element, since it shows that the initiatives mentioned earlier are not just mirrors of academic activities that ’make it visible’, but it also enable access from different social actors towards local knowledge (collaborative initiatives of citizen science, interaction between industry and academics, etc.), and it allows more rapid advancement in addressing local and Latin American needs in general. To achieve this, it is urgent that Chile follows the countries (Mexico, Argentina and Peru) that have established mandatory OA policies for their own national academic research.

## Data availability

No data are associated with this article.
