# Supplementary material for: Repositories for academic products/outputs: Latin American and Chilean visions
Source: F1000Res. 2019 Aug 28;8:1517. [Version 1] doi: 10.12688/f1000research.19976.1 (PMC6820816; doi:10.12688/f1000research.19976.1)
Supplement: Peer review history

# Peer review: Reviewer response for version 1

**DOI:** 10.5256/f1000research.21924.r53090

**Published:** 2019-10-28

**Authors:** Bianca Kramer

**Affiliations:** 1 Utrecht University Library, Utrecht University, Utrecht, The Netherlands

This article presents a view on open access and the role of repositories from a Latin American perspective, focusing on open access not only as a way to make scientific and scholarly publications more accessible, but also as part of aiming for more reproducible research. 

 It offers a discussion on goals of OA and reproducibility and places this in the context of institutional and government policies and practices, and the availability and usage of infrastructure in the context of Latin America, and Chili specifically. 

 As such, it provides an important point of view.

 I especially commend the authors for including Spanish-language literature in addition to English-language references.

 I agree with the first reviewer the paper could benefit from a tighter structure, removing some duplicate paragraphs, and fixing some missing and duplicate references.

 Regarding structure, I would suggest to make the aims of open access and reproducibility (in the context of Latin America) central to the paper, introducing them at the start and placing the discussion on OA definitions, policies, available infrastructure and recommendations in that context. 

 But this is just one suggestion, the structure suggested by the first reviewer is another one. 

 The paper mentions the relevance of OA and reproducibility for both natural and social sciences, as well as humanities. Building on that and using the references already provided, some discussion on the differences between these main disciplines in how these topics are considered could be a useful addition to the paper.  

 Beyond these main points, I have made a detailed annotation of the paper, including comments and suggestions (without the expectation they should all be met), as well as flagging some copy-editing issues using Hypothes.is. These are accessible here: https://hypothes.is/groups/4XjjYbQ1/f1000research-review or here.

I confirm that I have read this submission and believe that I have an appropriate level of expertise to confirm that it is of an acceptable scientific standard.

# Peer review: Reviewer response for version 1

**DOI:** 10.5256/f1000research.21924.r53091

**Published:** 2019-09-17

**Authors:** Claudio Gutierrez

**Affiliations:** 1 IMFD Chile and Department of Computer Science, University of Chile, Santiago, Chile

The article is mostly a survey (with a discussion) of the state of Open Access Policies in Latin America and in Chile.

 This is a relevant topic on which little systematic literature exists. Thus it is an important scientific contribution that deserves to be published.

 The material of the article is interesting, the authors have done a lot of work compiling sources and developing a map of the realm of Open Access in Latin America and in Chile.

 I only have formal observations, mainly regarding the organization of the paper that I will list below.

 A. I suggest to organize the material according to the three main topics treated (hopefully in this order) that currently are spread and mixed in different parts of the paper:

1. Open Access Policies and notions (varieties of OA, reproducibililty, legal issues, etc.). (page 3, page 7, page 9)
2. OA in Latin America (end of page 4, page 5, part of page 7, parts of page 9)
3. OA in Chile (page 4, page 6, second column of page 8, page 9)

B. I would develop a small taxonomy of OA policies and varieties and explain them. Not all readers are OA experts. If possible, explain relations among them, etc. Something like the description you did for Latin American OA initiatives.

 C: Revise repetitions throughout the paper. Example: In page 3:

- first paragraph of 1st column is repeated as fourth in second column
- last paragraph first column repeated in first three in second column

D. The readers of the paper are not only Chileans. Hence, do not write "at national level" but "at Chilean level", etc.

 E. Check details of form:

- be consistent when enumerating (capital o not capital letters)
- there is a [?] reference in page 9
- subtitles require to be more systematic

I approve this paper under the condition that the authors follow the corrections indicated.

I confirm that I have read this submission and believe that I have an appropriate level of expertise to confirm that it is of an acceptable scientific standard.
